# “Effect of post-kidney transplant diabetes mellitus on long-term outcomes in a cohort of pediatric kidney transplant recipients from 2005 to 2022.” Survival analysis

**DOI:** 10.1136/bmjpo-2024-002710

**Published:** 2024-12-24

**Authors:** Maria Alejandra Calvo-Herrera, Angelica Maria Serna-Campuzano, María Carolina Isaza-Lopez, Esteban Villegas-Arbeláez, Luisa Fernanda Rojas-Rosas, Lina Maria Serna-Higuita, Carolina Lucia Ochoa-García

**Affiliations:** 1 Pediatric Department, University of Antioquia, Medellin, Colombia; 2 Department of Nephrology, Hospital Pablo Tobón Uribe, Medellin, Colombia; 3 University Hospital San Vicente de Paul, Medellin, Colombia; 4 Universidad Pontificia Bolivariana, Medellin, Antioquia, Colombia; 5 Universidad CES Clinica CES, Medellin, Antioquia, Colombia; 6 Hospital Universitario General de Medellin, Medellin, Colombia; 7 Corporación Universitaria Remington, Medellin, Colombia; 8 Department of Clinical Epidemiology and Applied Biostatistics, University Hospital Tübingen, Tubingen, Germany

**Keywords:** Child Health, Nephrology, Mortality, Endocrinology

## Abstract

**Background:**

Post-transplantation diabetes mellitus and carbohydrate intolerance (PTDM/iCHO) are complications following solid organ transplantation, which significantly increases the risk of graft loss and mortality. However, data concerning long-term outcomes in paediatric kidney transplant recipients with PTDM/iCHO are scarce. This study aimed to evaluate the risk of graft loss in paediatric kidney transplant recipients with PTDM or iCHO compared with non-PTDM/iCHO.

**Methods:**

The study cohort included patients aged <18 who underwent a kidney transplant in a transplant centre from 2005 to 2022. The primary outcome was graft survival loss; secondary outcomes were acute rejection, renal function and mortality. Cumulative incidence of graft loss and acute rejection was estimated, considering death a competing risk. Fine and Gray’s proportional subdistribution hazard model was used to analyse the effect of PTDM/iCHO status on the event.

**Results:**

Seventy-six paediatric kidney transplant recipients were included. The incidence of PTDM and iCHO was 6.6% and 9.2%, respectively. Patients with PTDM/iCHO had a significantly higher cumulative graft loss incidence than those without (34.4% vs 13.9% at 36 months, p<0.008). Multivariable analysis revealed a threefold increased risk of graft loss in patients with PTDM/iCHO (HR_adjusted_ 3.33, 95% CI 1.19 to 9.30, p=0.022). PTDM/iCHO was associated with a higher incidence of acute rejection (33.3% vs 14.5% at 1 year, p=0.025). Patients with PTDM/iCHO also exhibited significantly worse eGFR at all time points compared with patients without PTDM/iCHO (p=0.036)

**Conclusion:**

Patients with PTDM and iCHO had a higher risk of graft loss and lower renal function in paediatric kidney transplant recipients. This justifies close monitoring of metabolic complications in these patients.

**WHAT IS ALREADY KNOWN ON THIS TOPIC**

**WHAT THIS STUDY ADDS**

- The presence of PTDM/iCHO resulted in significantly more graft loss and lower renal function compared with patients without this complication.

**HOW THIS STUDY MIGHT AFFECT RESEARCH, PRACTICE OR POLICY**

- Our results also suggest that PTDM/iCHO is associated with worse outcomes, including graft loss and less overall survival.

## Introduction

 Kidney transplantation (KT) is currently the therapy of choice for children with end-stage renal disease^1^ because of improvements in quality of life and survival compared with dialysis.^2^,^5^ Data from the Organ Procurement and Transplant Network (OPTN) show that 4.8% of the solid organ transplantation performed in the USA in 2019 were in patients under 18 years.^1^ According to the 2019 annual Colombian report of the Donation and Transplantation Network, 4.4% of kidney transplants performed in 2021 were in patients under the age of 18.^6^

With the development of medical technology, excellent surgical skills and effective immunosuppressive medications, the incidence of short-term postoperative complications, such as acute rejection, has been significantly reduced.^1 2^ However, there is a large discrepancy between short-term and long-term post-transplant outcomes, as the latter remains less than optimal.^2^,^4^ Post-transplantation diabetes mellitus (PTDM) and carbohydrate intolerance (iCHO) are important complications after KT.^37^,^9^ The incidence of PTDM is 4%–25% in adults and 3%–20% in children and adolescents.^19^,^11^ Although PTDM in the paediatric population remains less prevalent than in the adult population, these conditions are especially important given that iCHO and PTDM play a pivotal role in the development and acceleration of cardiovascular complications.^9^ ^12^ Different studies have shown that PTDM or iCHO negatively impacts long-term graft and overall survival.^112^,^16^

Published data concerning PTDM in paediatric recipients are limited,^2 7 17^ and the association between diabetes and mortality in children after KT is inconclusive.^1 17^ Although cardiovascular mortality is high in paediatric patients,^18^ there is limited evidence on whether optimising glycaemic control in PTDM in the paediatric population improves outcomes.^18^ This study aims to determine the incidence of PTDM among paediatric KT patients at a tertiary care child′s hospital from 2005 to 2022 and identify the impact on graft loss, acute rejection and overall survival.

## Methods

### Study design and population

This single-centre retrospective cohort study included paediatric patients aged 0–18 years who received a KT at the Pablo Tobón Uribe Hospital (HPTU) between January 2005 and December 2022 and had fasting glucose values 42 days after KT. Exclusion criteria included previous diagnoses of diabetes mellitus or impaired glucose tolerance, multiple organ transplants and those with a follow-up time of less than 1 year.

### Data source

Data were obtained retrospectively from the Institutional Registry of Renal Transplant Patients at HPTU in Medellín, Colombia. Additional clinical data not available in that register were extracted from the medical records.

### Data collection

Recipient-related clinical data and relevant transplant donor characteristics were collected. Laboratory investigations included oral glucose tolerance test, fasting glucose test, glycosylated haemoglobin and creatinine.

The diagnosis of PTDM and iCHO in this study is based on the diabetes definition by the American Diabetes Association criteria in the absence of evidence of pretransplantation DM.^19^ The diagnosis was based on the following criteria: (1) patients who had fasting plasma glucose concentration (FPG) >126 mg/dL (7 mmol/L) or non-fasting plasma glucose concentration ≥200 mg/dL (11.1 mmol/L) were defined as having PTDM. (2) FPG levels between 100 and 125 mg/dL and second-hour glucose values of OGTT between 140 and 199 mg/dL were defined as having impaired carbohydrate tolerance (iCHO). (3) Haemoglobin A_1_C >6.5% is considered PTDM, and values between 5.6% and 6.4% indicate iCHO, and (4) the absence of other related causes, such as stress hyperglycaemia, high esteroid doses or use of diabetogenic medications other than calcineurin inhibitors (CNI). The status of these two outcomes (PTDM or iCHO) was evaluated ≥42 days after transplantation.^20^

### Outcomes

The primary outcome of this study was the presence of renal graft loss, defined as reinitiation of dialysis, receiving a new transplant, or having a glomerular filtration rate of less than 15 mL/m^2^/min for 3 months.^21^ The secondary outcome was biopsy-proven acute rejection (BPAR), defined as creatinine elevation accompanied by pathological evidence of rejection. Other secondary endpoints included overall survival and renal function. Renal function assessment was performed by calculating the glomerular filtration rate (mL/min/1.73 m^2^) according to the Schwartz formula.^22^

### Sample size calculation

A post-hoc power calculation was done to estimate the minimum effect size of the primary outcome A sample size calculation was performed to assess the minimum number of patients needed to detect a 25% difference in graft loss presence (graft loss at 24 months 9.5%^23^ vs 34.5% in patients with diabetes or iCHO). The calculation was done using PASS 2020 software. A sample size of 80 patients achieves 80% power and a 5% alpha to detect a difference between hazards of 0.162 (exponential parameter of the control group, λ_1_ of 0.0499 and an exponential parameter of the control group, λ_2_, of 0.211 with a constant HR of 4.2).

### Statistical analysis

Nominal variables were expressed as frequency and percentage. Quantitative variables were reported with mean and SD or median and IQR, depending on the distribution of variables for each group. The assumption of normal distribution was evaluated graphically using histograms, Q–Q plots, box plots, skewness and kurtosis. Subsequently, the evaluated cohort was divided into two groups: group 1, patients without PTDM/iCHO, and group 2, patients with PTDM/iCHO. Bivariate analyses were performed using the appropriate χ^2^ test or Fisher’s exact test. Independent-sample t-tests were used to compare numerical variables that were approximately normally distributed, while Mann–Whitney tests were used to evaluate skewed variables.

The association between PTDM or iCHO and graft loss of BPAR was assessed using survival analysis. The Kaplan-Meier method was used to calculate the cumulative incidence (CI) of PTDM and iCHO. Univariable and multivariable Sub-distribution Cox Proportional-Hazard models of Fine and Gray were used to compare exposure groups (PTDM/iCHO). For this analysis, the event of interest was graft loss, and the follow-up time was the period from KT to the occurrence of the event (graft loss). Patients who did not experience the event were considered censored. Finally, death was considered a competing risk. The assumption of proportional hazards was evaluated using Schoenfeld residuals. Finally, different nested models were compared using the log-likelihood test, and the goodness of fit of these models was evaluated using the −2LL statistic. In the final model, β coefficients, HRs, CIs and the statistical significance of variables included in the final model were estimated. Using a linear mixed model, the changes in the eGFR over time were estimated, with time points being nested within the participants and including a random intercept for subjects.

IBM SPSS V.29.0 (IBM) and R software V.4.2 were used for data analysis. All reported p values are two-tailed, and the significance level was set at ≤0.05.

The results were reported using the Strengthening the Reporting of Observational Studies in Epidemiology statement recommendations for cohort studies.

### Handling of missing data

Multiple imputation was performed to avoid excluding patients with missing data.^4^ A total of 50 imputations were performed and the final estimates were obtained following Rubin’s rules.

### Patient and public involvement

There was no patient or public involvement in the design, or conduct, or reporting, or dissemination plans of our study. The data were collected with ethic approvals described under ethics approval section in this paper.

## Results

**Figure 1:**
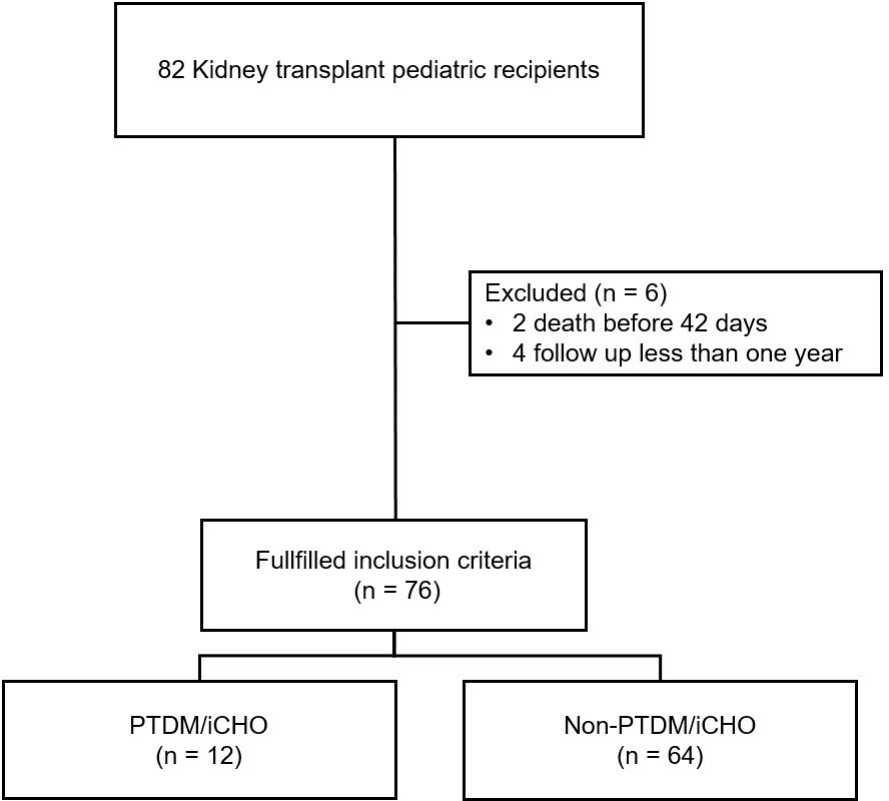
Flow chart total cohort. PTDM, post-transplantation diabetes mellitus; iCHO, carbohydrate intolerance.

Among 82 children who received a KT between January 2015 and December 2022, six patients were excluded. In total, 76 patients were included (figure 1). There were 44 (57.9%) male. The mean age at the moment of KT was 13.2 (±4.4) years, and the median follow-up period was 6.11 (p25-75: 2.7–9.7) years. The baseline demographic characteristics and clinical data are shown in table 1. The maintenance immunosuppression protocol for all patients consisted of CNIs (cyclosporine or tacrolimus), antiproliferative agents (azathioprine or mycophenolic acid derivate) and steroids.

**Table 1:** Baseline characteristics (n=76)

	N	With PTDM/iCHO(n=12)	Without PTDM/iCHO (n=64)	P value	Total cohort (n=76)
Gender					
Male n (%)	76	6 (50.0%)	38 (59.4%)	0.78^Chi^	44 (57.9%)
Female n (%)		6 (50.0%)	26 (40.6%)		32 (42.1%)
Age (years)	76				
Mean (±SD)		13.5 (3.50)	13.1 (4.58)	0.74^TT^	13.2 (4.41)
Weight	61				
Mean (±SD)		37.8 (13.2)	37.7 (16.2)	0.97^TT^	37.7 (15.7)
Body mass index at transplant					
Mean (±SD)	69	19.0 (4.49)	18.2 (4.24)	0.60^TT^	18.3 (4.25)
Primary renal disease					
Glomerulopathies	76	3 (25%)	23 (35.9%)		26 (34.2%)
Unknown kidney disease		4 (33,3%)	12 (18.8%)		16 (21.1%)
Obstructive uropathies		4 (33.3%)	17 (26.6%)		21 (27.6%)
Renal dysplasia		1 (8.3%)	7 (10.9%)		8 (10.5%)
Alport syndrome		0 (0%)	3 (4.7%)		3 (3.9%)
Others		0 (0%)	2 (3.1%)		2 (2.6%)
History of hypertension					
No n (%)	76	11 (91.7%)	48 (75.0%)	0.37^FT^	59 (77.6%)
Yes n (%)		1 (8.3%)	16 (25.0%)		17 (22.4%)
History of cerebrovascular disease					
No n (%)	76	12 (100%)	61 (95.3%)	0.99^FT^	73 (96.1%)
Yes n (%)		0 (0%)	3 (4.7%)		3 (3.9%)
History of hyperlipidaemia					
No n (%)	76	12 (100%)	62 (96.9%)	0.99^FT^	74 (97.4%)
Yes n (%)		0 (0%)	2 (3.1%)		2 (2.6%)
Pretransplant dialysis					
No n (%)	76	2 (16.7%)	14 (21.9%)	0.13^FT^	16 (21.1%)
Peritoneal n (%)		9 (75.0%)	29 (45.3%)		38 (50.0%)
Haemodialysis n (%)		1 (8.3%)	21 (32.8%)		22 (28.9%)

.χ, chi-squared test; FT, exact Fisher testiCHOcarbohydrate intolerancePDTMpost-transplantation diabetes mellitusTT, Student test

**Figure 2:**
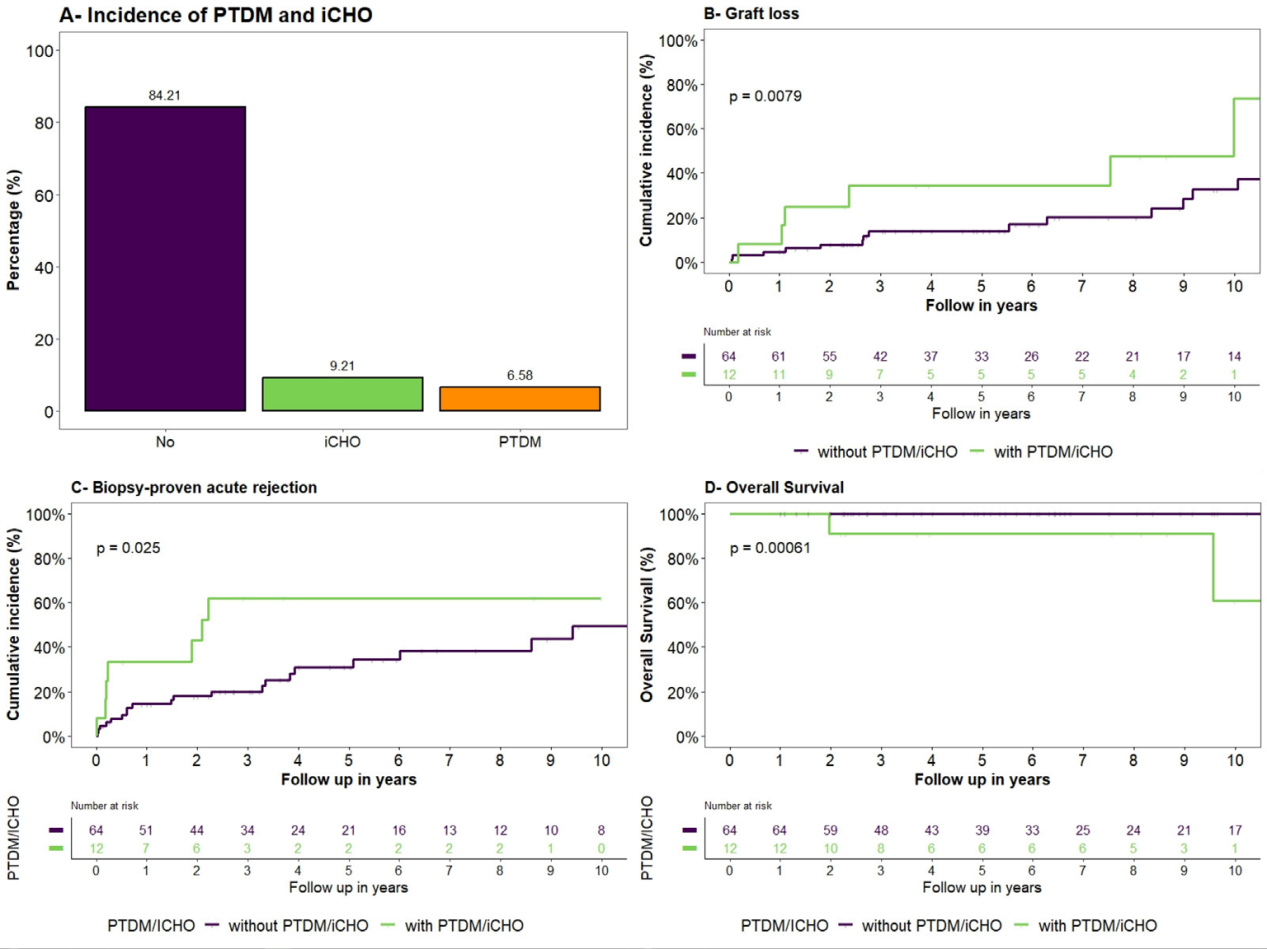
Incidence of post-transplantation diabetes mellitus (PTDM) and carbohydrate intolerance (iCHO) and Kaplan-Meier curves: (A) shows the incidence of PTDM and iCHO. (B) Cumulative incidence of the primary outcome graft loss and (C) the secondary outcome biopsy-proven acute rejection according to the presence of PTDM/iCHO. (D) Kaplan-Meier curves for overall survival.

Five patients (6.6%) were diagnosed with PTDM and 7 (9.2%) iCHO (figure 2A). The median time to PTDM/iCHO diagnosis was 1.95 (IQR=0.83–4.9) years. Baseline characteristics were similar in both cohort groups (tables1  2).

**Table 2:** Pretransplant characteristics (n=76)

	N	With PTDM/iCHO, yes(n=12)	Without PTDM/iCHO (n=64)	P value	Total cohort (n=76)
Donor age (years)					
Mean (±SD)	74	28.7 (11.6)	25.0 (12.1)	0.34^TT^	25.6 (12.1)
Creatinine donor					
Mean (±SD)	73	0.83 (0.25)	0.82 (0.36)	0.87^TT^	0.82 (0.34)
Deceased donor					
Yes n (%)	76	12 (100%)	63 (98.4%)	0.99^FT^	75 (98.7%)
No n (%)		0 (0%)	1 (1.6%)		1 (1.3%)
First transplant					
Yes n (%)	76	11 (91.7%)	58 (90.6%)	0.99^FT^	69 (90.8%)
Not n (%)		1 (8.3%)	6 (9.4%)		7 (9.2%)
Cold ischaemia time (hours)					
Median (p25−75)	74	14.5 [10.7–17)	17.0(12, 21)	0.15^MM^	16.7(12 - 21)
Cytomegalovirus status					
IgG− IgM−	74	2 (18.2%)	17 (28.3%)	0.62^FT^	19 (26.8%)
IgG+ IgM−		9 (81.8%)	41 (68.3%)		50 (70.4%)
IgG− IgM +		0 (0%)	0 (0%)		0 (0%)
IgG+ IgM +		0 (0%)	2 (3.3%)		2 (2.8%)
Previous Hep C infection					
Yes n (%)	75	1 (8.3%)	0 (0%)		1 (1.3%)
No n (%)		11 (91.7%)	63 (100%)		75 (98.7%)
Induction therapy					
No n (%)	75	0 (0%)	6 (9.5%)	0.59^FT^	6 (8.0%)
Yes n (%)		12 (100%)	57 (90.5%)		69 (92.0%)
Induction therapy					
Thymoglobulin	75	3 (25.0%)	24 (39.3%)		27 (37.0%)
Alemtuzumab		3 (25.0%)	20 (32.8%)		23 (31.5%)
Basiliximab		4 (33.3%)	13 (21.3%)		17 (23.3%)
Daclizumab		1 (8.3%)	2 (3.3%)		3 (4.%)
Solumedrol		1 (8.3%)	2 (3.3%)		3 (4.1%)
Steroid therapy					
No n (%)	76	0 (0%)	0 (0%)		0 (0%)
Yes n (%)		12 (100%)	64 (100%)		76 (100%)
Immunosuppressive regimen					
MMF–TAC	76	6 (50.0%)	25 (39.1%)	0.94^FT^	31 (40.8%)
MMF–Cyc		0 (0%)	6 (9.4%)		6 (7.9%)
Aza–Cyc		1 (8.3%)	8 (12.5%)		9 (11.8%)
Aza–Tac		5 (41.7%)	25 (39.1%)		30 (39.5%)

.Aza, azathioprine; Chichi-squared testcyccyclosporineFTexact Fisher testiCHQcarbohydrate intoleranceMMFmycophenolate mofetilPTDMpost-transplantation diabetes mellitusTACtacrolimusTTStudent test

### Primary endpoint: graft loss

During a median follow-up of 6.1 years, 23 patients experienced graft loss, the most cause of graft loss was humoral rejection (15.8%), acute rejection (5.3%), disease recurrence (3.9%) and Bk virus infection (2.6%). CI of graft loss for each cohort group are shown in figure 2B. The CI of graft loss at 12, 24 and 36 months in the group with PTDM/iCHO was 8.3%, 25% and 34.4%, respectively, compared with 4.7%, 8.0% and 13.9% in the group without PTDM/iCHO, respectively (p=0.008). Table 3 shows the results of the univariable and multivariable subdistribution Cox hazard models, which provide evidence of the association between PTDM/iCHO and graft loss. The association remained significant after adjustment for age, dialysis pretransplant, induction therapy and competing risks (HR_adjusted_ 3.33, 95% CI 1.19 to 9.30 p=0.022).

**Table 3:** Univariable and multivariable analysis, Fine–Gray Subdistribution HR, outcome graft loss

	Univariable analysis			Multivariable analysis		
	HR	95% CI	P value	HR	95% CI	P value
Gender						
Male	1					
Female	1.72	0.77 to 3.85	0.19			
Age	1.10	0.99 to 1.20	0.051	1.08	0.96 to 1.20	0.19
BMI	1.09	0.99 to 1.21	0.08			
Comorbidity	1.09	0.43 to 2.77	0.85			
History of hypertension	0.46	0.14 to 1.49	0.19			
Previous dialysis	7.18	1.02 to 50.7	0.048	4.86	0.81 to 29.0	0.083
Creatinine values donor	2.08	0.81 to 5.28	0.12			
Cold ischaemia time	0.98	0.96 to 1.00	0.09			
Induction therapy	0.27	0.09 to 0.83	0.022	0.22	0.08 to 0.64	0.005
Maintenance therapy						
Aza – Cyc	1					
Aza – Tac	1.37	0.32 to 5.82	0.67			
MMF – Cyc	3.03	0.52 to 17.5	0.22			
MMF – Tac	1.53	0.35 to 6.76	0.57			
iCHO/PTDM	3.02	1.26 to 7.22	0.013	3.33	1.19 to 9.30	0.022

.Aza, azathioprine; BMIbody mass indexCyc, cyclosporine; iCHOcarbohydrate intoleranceMMF, mycophenolate mofetilPTDMpost-transplantation diabetes mellitusTactacrolimus

### Secondary endpoints

A total of 30 patients with BPAR were observed. The 1-year CI of BPAR after KT was 33.3% in the PTDM/iCHO and 14.5% in the group without PTDM/iCHO (p=0.025) (figure 2C). The number of T-cell-mediated BPAR graded higher than 1A was increased in transplant recipients with PTDM/iCHO compared with those patients without PTDM/iCHO (table 4). In the multivariate subdistribution Cox proportional hazards model, PTDM/iCHO (HR_adjusted_ 2.47 95% CI 0.94 to 6.46, p=0.07) (data not shown) was not a significant risk factor for BPAR.

**Table 4:** Severity of biopsy-proven acute rejection

Variable	With PTDM/iCHO yes (n=12)	Without PTDM/iCHO (n=64)	P value	Total cohort (n=76)
**Acute T-cell-mediated rejection**	**6** (**50%**)	**15** (**23.4%**)	**0.052^FT^**	**21**
1A	1 (8.3%)	8 (12.5%)		9
1B	3 (25%)	3 (4.7%)		6
2A	2 (16.7%)	3 (4.7%)		5
2B	0 (0%)	1 (1.6%)		1
3	0 (0%)	0 (0%)		0
**Acute antibody-mediated rejection**	**1** (**8.3%**)	**8** (**12.5%**)		**9**
**Non-BPAR**	**5** (**41.7%**)	**41** (**64.0%**)		**46**

BPARbiopsy-proven acute rejectionFTexact Fisher testiCHOcarbohydrate intolerancePTDMpost-transplantation diabetes mellitus

In total, two deaths were observed. One died with a functioning graft (death was due to COVID-19 infection), and one died after graft loss. The Kaplan-Meier analysis showed that the survival curve of patients without PTDM/iCHO differed from that of patients with PTDM/iCHO (p*<*0.001) (figure 2D).

### Glomerular filtration rate

**Figure 3:**
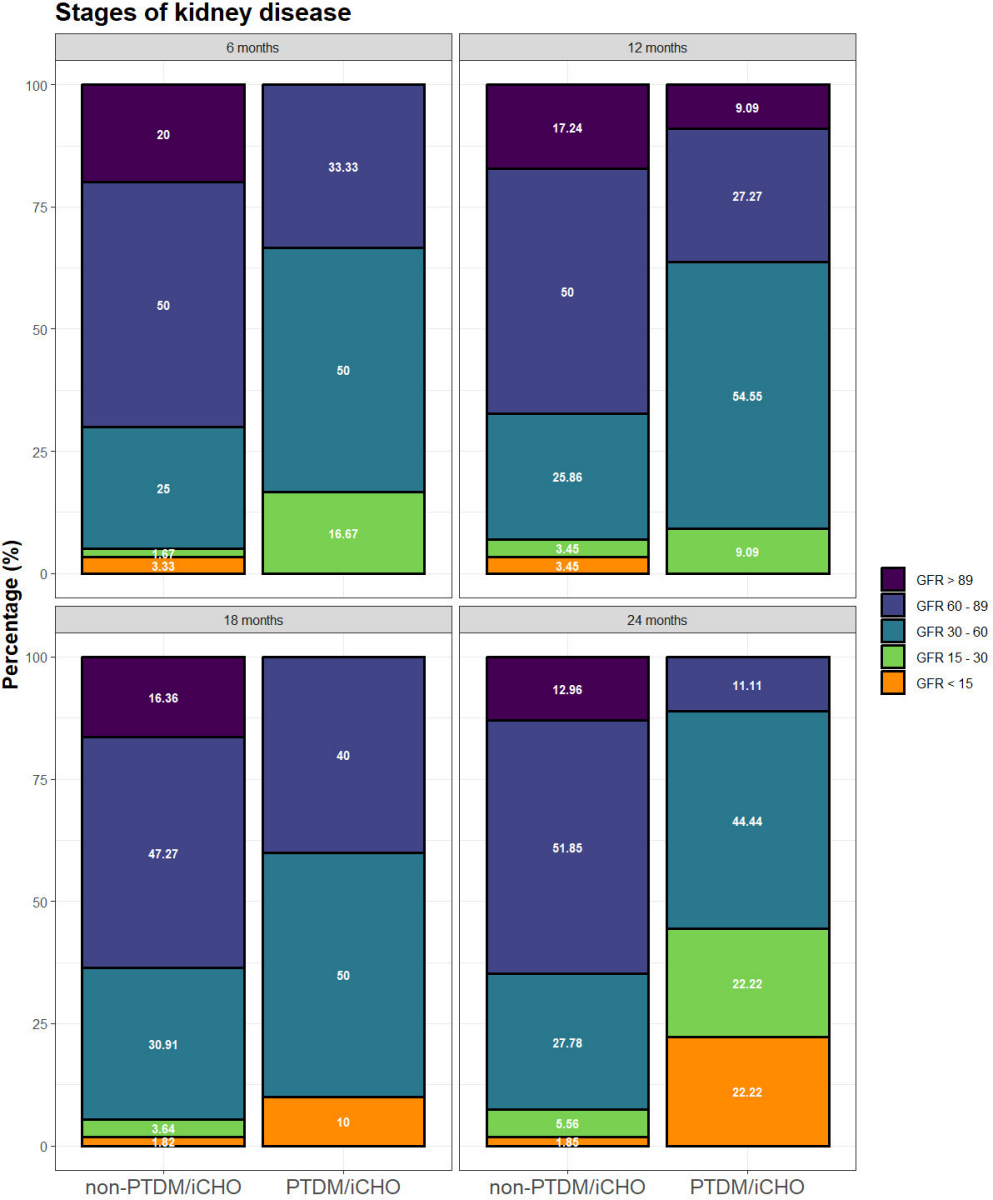
Glomerular filtration rate (GFR) during follow-up and stratified by categories estimated glomerular filtration rate (eGFR): mL/min/1.73 m^2^. Chronic kidney disease classification is as follows: stage 1 eGFR >89, stage 2 eGFR 60–89, stage 3 eGFR 30–59, stage 4 eGFR 15–30, stage 5 eGFR <15. iCHO, carbohydrate intolerance; PTDM, post-transplantation diabetes mellitus.

At 2 years post-transplant, the mean eGFRs (IQR) of the non-PTDM/iCHO group was 68.1 (±22.7) mL/min/1.73 m^2^ (n = 54) and 34.9 (±22.1) (n = 9) in the group with-PTDM/iCHO. We found that eGFR shows a decrease in allograft function during the follow-up (β=−0.36, p<0.001) (table 5). Patients with PTDM/iCHO presented significantly worse graft function at each time point than non-PTDM/iCHO (p=0.036) (table 5). However, there were no statistically significant differences in the change in eGFR over time in both groups (p=0.293). When grouping patients by chronic kidney disease (CKD) stage, more patients in the group PTDM/iCHO present CKD stages 4 and 5 (eGFR <30 mL/min/1.73 m^2^) than in those without PTDM/iCHO (figure 3).

**Table 5:** Glomerular filtration rate during the follow-up, linear mixed model with random intercept

	Estimate (β)	df	95% CI	P value
iCHO/PTDM, yes	−14.4	91.4	−27.6 to −1.17	0.036
Time	−0.36	370.8	−0.47 to −0.26	<0.001
Interaction time: iCHO/PTDM	−0.15	371.8	−0.43 to 0.13	0.293

.df, degrades of freedomiCHOcarbohydrate intolerancePTDMpost-transplantation diabetes mellitus

## Discussion

PTMD is associated with adverse patient outcomes, including cardiovascular disease, graft loss and mortality.^112^,^15 19 24^ However, published data concerning PTDM in paediatric recipients are limited, and our knowledge is gleaned mainly from research on adult patients. Our study aims to assess the incidence of PTDM and its impact on graft loss among a cohort of 76 paediatric kidney transplant recipients. The main findings of this retrospective study are that the presence of PTDM/iCHO increases the risk of graft loss and reduced kidney function. This is the first observational study performed in the Colombian paediatric population that evaluates the incidence of PTDM and iCHOS and its implications for graft loss, acute rejection and diminution of the eGFR.

In the current study, the incidence of PTDM/iCHO was 15.8%, and 6.6% of patients had PTDM. Our findings are similar to previously reported PTDM rates, ranging from 7% to 13%.^25 26^ iCHO was detected in 9.2% of patients, which is lower than in other studies where the incidence of abnormal glucose metabolism was up to 71.4%.^3 26^ Although this incidence is less than that was described by previous studies, it is still important as iCHO is the strongest predictor for future development of PTDM.^15 17^ A study in paediatric transplant recipients found that iCHO increased the risk of PTDM threefold in the first year post transplant. (reference: 14). Therefore, identifying iCHO early and offering early intervention may positively impact the prevention of future risks of PTDM.

Our study showed higher graft loss in patients with PTDM/iCHO compared with the non-PTDM/iCHO group, which persists even after adjusting the multivariable model with a threefold increased risk of graft loss in patients with PTDM/iCHO. Previous research has failed to find this association in paediatric transplant recipients. In the study of Kuo *et al*,^27^ which included 2726 kidney transplant recipients, the 2-year graft loss rates were similar between those with PTDM and non-PTDM.^27^ Similarly, in a US register (OPTN/United Network for Organ Sharing and the United States Renal Data System (USRDS) databases) analysis of KT in 1995–2004, Burroughs *et al* failed to identify an association between the development of PTDM and graft failure over 3-year follow-up.^28^ This discrepancy might have several possible explanations, including variable follow-up time after KT (2–3 years vs 5.5 years in our study), heterogeneous cohorts using different definitions of PTDM and temporal trends in post-transplant care and immunosuppressive medications.^17^

Our study reported more rejection episodes in patients with PTDM/iCHO than without PTM/iCHO, mainly secondary to T-cell mediated BPAR graded higher than 1A. Nevertheless, non-statistical significance was reached in the multivariable model. This finding is consistent with previous studies. For example, Al-Uzri *et al* found that the presence of PTDM led to a borderline statistical increase in the risk of acute rejection (p=0.058).^29^

The association between PTDM and mortality is conflicting. In previous studies, PTDM was associated with an increased risk of death.^12 17^ In contrast, another study involving 2168 paediatric renal transplant recipients found no significant association between diabetes and mortality (HR 1.51; 95% CI 0.57 to 3.99).^28^ Our study found that the 2-year cumulative overall survival rate was lower in the PTDM/iCHO group (90.9% vs 100% in the group without PTDM/iCHO, p<0.001). However, due to the small sample size, we cannot provide precise mortality estimates.

PTDM arises from an interaction between a combination of direct toxic effects on pancreatic β cells which lead dysfunction and insulin resistance in peripheral tissues with superimposed immunosuppression, which accelerates pre-existing damage.^19 30^ Therefore, early identification of those patients with PTDM is critical to ensure better long-term outcomes^19^ by addressing modifiable risk factors and emphasising lifestyle modification, including dietary modification and physical exercise,^15^ as well as effectively managing diabetes using pharmacological and non-pharmacological strategies.^19^ Otherwise, considering the diabetogenic effect of some immunosuppressive medications,^30^ immunosuppressant regimens need to be individualised, balancing the risk of post-transplant hyperglycaemia or diabetes against the risk of graft rejection.^14 19^

### Strengths and limitations

Our study has several limitations. First, it is a retrospective design. Second is the risk of misclassification since the OGTT, which has greater sensitivity to detect PTDM and iCHO, was not used to screen patients.^3^ Third, the small sample size may cause our study to be underpowered in detecting some predictors, as evidenced in the wider confidence intervals. Fourth, due to the small number of events and the small sample size, we opted for a model with a limited number of variables. Selecting the optimal model in the presence of multiple potential confounding variables is inherently difficult, and it is possible that some important variables related to the event were not included. Larger studies are needed to further validate these results and provide more robust conclusions.

Despite these limitations, our study used a standard definition of PTDM and adequate statistical methods to avoid bias; in addition, we performed the study in a high-complexity centre with considerable experience and where many patients are treated. That is why we consider that these findings may represent the behaviour of this pathology in other centres.

## Conclusion

In conclusion, our study suggests that PTDM/iCHO in paediatric recipients is associated with worse outcomes, including graft loss and less overall survival. As PTDM/iCHO is modifiable, early identification and timely intervention may positively impact the long-term outcomes of paediatric KT recipients.

## Data Availability

Data are available upon reasonable request.
