# Supplementary material for: “Effect of post-kidney transplant diabetes mellitus on long-term outcomes in a cohort of pediatric kidney transplant recipients from 2005 to 2022.” Survival analysis
Source: BMJ Paediatr Open. 2024 Dec 24;8(1):e002710. doi: 10.1136/bmjpo-2024-002710 (PMC11683984; doi:10.1136/bmjpo-2024-002710)
Supplement: Supplementary file 1 [file bmjpo-2024-002710.reviewer_comments.pdf]

PEER REVIEW HISTORY

BMJ Paediatrics Open publishes all reviews undertaken for accepted manuscripts. Reviewers are asked to complete a checklist review form and are provided with free text boxes to elaborate on their assessment. These free text comments are reproduced below.

ARTICLE DETAILS

**TITLE (PROVISIONAL)**	"Effect of post-kidney transplant diabetes mellitus on long-term outcomes in a cohort of pediatric kidney transplant recipients from 2005 to 2022." Survival analysis.
**AUTHORS**	Maria, Calvo-Herrera; Angelica, Serna-Campuzano; María, Isaza-Lopez; Villegas-Arbeláez, Esteban; Rojas Rosas, Luisa; Serna-Higuita, Lina Maria; Carolina, Ochoa-García

VERSION 1 - REVIEW

**REVIEWER NAME**	***Lars Pape***
**REVIEWER AFFILIATION**	
**REVIEWER CONFLICT OF INTEREST**	
**DATE REVIEW RETURNED**	20-May-2024

**GENERAL COMMENTS**	The authors have well implemented the editors' comments.

**REVIEWER NAME**	***Sukanya Govindan***
**REVIEWER AFFILIATION**	Dr Mehta's Hospitals Pvt Ltd, Pediatric nephrology
**REVIEWER CONFLICT OF INTEREST**	
**DATE REVIEW RETURNED**	16-Nov-2024

**GENERAL COMMENTS**	This is a very relevant study and the authors need to be commended for looking at the long term graft survival in pediatric kidney transplant recipients in Colombia.<br><br>There are a few changes advised that would make the present study more useful to the readers.<br><br>1) Introduction: ESRD - should be expanded as end stage renal disease.<br>2) One of the major gaps in the reported data is that the authors fail to address many confounders for graft loss and development of PTDM - some of them include CNI trough levels, steroid use (higher use for patients with rejection), Hepatitis C infection all of which can be associated with both PTDM and graft survival or loss.<br>3) Also increased risk of infections would be considered a major risk factor for allograft dysfunction and patient survival in patients with PTDM.<br><br>4) Authors should clearly state all the variables that were adjusted in the multivariate analyses before concluding that PTDM is independently associated with increased graft loss.

	I suggest that the authors perform re- analysis with adjustment for confounders and present information on multivariate data analysis for review.

VERSION 1 – AUTHOR RESPONSE

Reviewer: 1

Comments to the Author

The authors have well implemented the editors' comments.

Author's reply: thank you very much for the comment.

Reviewer: 2

Dr. Sukanya Govindan, Dr Mehta's Hospitals Pvt Ltd

Comments to the Author

This is a very relevant study and the authors need to be commended for looking at the long-term graft survival in pediatric kidney transplant recipients in Colombia. There are a few changes advised that would make the present study more useful to the readers.

1) Introduction: ESRD - should be expanded as end stage renal disease.

Author reply: With thanks to the reviewers we are change the definition of ESRD.

2) One of the major gaps in the reported data is that the authors fail to address many confounders for graft loss and development of PTDM - some of them include CNI trough levels, steroid use (higher use for patients with rejection), Hepatitis C infection all of which can be associated with both PTDM and graft survival or loss.

Author´s reply: we thank the reviewer for this comment. We absolutely agree that those variables should be included into the multivariable model. Unfortunately, there is no available information in the database about the CNI through levels. Regarding steroids therapy, all patients in the cohort received low-doses steroids, however, the specific steroid dosages administered during the follow up were not recorded. Finally, only one patient has history of Hepatitis C infection, which is too low number to be included in a multivariable model. However, we agree that is a limitation of our study, and therefore, we have made amendments to the limitations section to address the reviewer's comment.

We would also like to emphasize that we checked that the hyperglycemia in these patients was not caused by other factors, such as hyperglycemia due to stress, high doses of steroids or the use of diabetogenic drugs other than calcineurin inhibitors.

3) Also increased risk of infections would be considered a major risk factor for allograft dysfunction and patient survival in patients with PTDM.

Author´s reply: we thank the reviewer for this comment. We absolutely agree that the presence of infections increase the risk of allograft dysfunction and negatively impacts patient survival. Unfortunately, our database does not include information on infection post transplantation. Moreover, the risk of infection could act as a mediator variable, wherein the relationship between the independent variable (PTDM) and the dependent variable (Graft loss) is influenced through another variable (infection) as an intermediary. However, the statistical model proposed for our analysis is not ideally for assessing mediation effect.

4) Authors should clearly state all the variables that were adjusted in the multivariate analyses before concluding that PTDM is independently associated with increased graft loss.

Author’s reply – Thank you. we thank the reviewer for this suggestion, however we must emphasize that our cohort records a total of 23 Events (Graft loss) and only 2 deaths). This limited number imposes significant limitations when considering the inclusion of multiple variables in a multivariate model and in our case, a model including competing risks. Models with fewer than 5 events per predictor variable are susceptible to increased bias and variability, leading to unreliable confidence intervals and convergence issues that affect model accuracy (ref: Vittinghoff E, McCulloch CE. Relaxing the rule of ten events per variable in logistic and Cox regression. Am J Epidemiol. 2007 Mar 15;165(6):710-8. doi: 10.1093/aje/kwk052. Epub 2006 Dec 20. PMID: 17182981). In this specific analysis, the multivariable model performed has already included four variables, exceeding the typical threshold for including variables in a model that is required for accurate estimation of regression coefficients and standard errors (Austin PC, Allignol A, Fine JP. The number of primary events per variable affects estimation of the subdistribution hazard competing risks model. J Clin Epidemiol. 2017 Mar;83:75-84. doi: 10.1016/j.jclinepi.2016.11.017. Epub 2017 Jan 12. PMID: 28088594). However, we agree that is a limitation of our study, and therefore, we have made amendments to the limitations section to address the reviewer’s comment
